# PTEN inhibits replicative senescence‐induced MMP‐1 expression by regulating NOX4‐mediated ROS in human dermal fibroblasts

**DOI:** 10.1111/jcmm.13220

**Published:** 2017-05-30

**Authors:** Eun‐Mi Noh, Jeong‐Mi Kim, On‐Yu Hong, Hyun‐Kyung Song, Jong‐Suk Kim, Kang‐Beom Kwon, Young‐Rae Lee

**Affiliations:** ^1^ Center for Metabolic Function Regulation Wonkwang University School of Medicine Iksan City Jeonbuk South Korea; ^2^ Department of Biochemistry Institute of Medical Science Chonbuk National University Medical School Jeonju South Korea; ^3^ Department of Korean Physiology Wonkwang University School of Korean Medicine Iksan City Jeonbuk South Korea; ^4^ Department of Oral Biochemistry Institute of Biomaterials, Implant, School of Dentistry Wonkwang University Iksan City Jeonbuk South Korea

**Keywords:** skin ageing, PTEN, NADPH oxidase‐4, reactive oxygen species, MMP‐1

## Abstract

The biological function of NADPH oxidase (NOX) is the generation of reactive oxygen species (ROS). ROS, primarily arising from oxidative cell metabolism, play a major role in both chronological ageing and photoageing. ROS in extrinsic and intrinsic skin ageing may be assumed to induce the expression of matrix metalloproteinases. NADPH oxidase is closely linked with phosphatidylinositol 3‐OH kinase (PI3K) signalling. Protein kinase C (PKC), a downstream molecule of PI3K, is essential for superoxide generation by NADPH oxidase. However, the effect of PTEN and NOX4 in replicative‐aged MMPs expression has not been determined. In this study, we confirmed that inhibition of the PI3K signalling pathway by PTEN gene transfer abolished the NOX‐4 and MMP‐1 expression. Also, NOX‐4 down‐expression of replicative‐aged skin cells abolished the MMP‐1 expression and ROS generation. These results suggest that increase of MMP‐1 expression by replicative‐induced ROS is related to the change in the PTEN and NOX expression.

## Introduction

Skin ageing can be divided into intrinsic or chronologic ageing, which is the process of senescence that affects all body organs, and extrinsic ageing (photoaging), which occurs as a consequence of exposure to environmental factors 1, 2. ROS, primarily arising from oxidative cell metabolism, play a major role in both chronological ageing and photoaging 3. ROS in extrinsic and intrinsic skin ageing may induce the transcription factors NF‐κB and c‐Jun *via* mitogen‐activated protein kinases (MAPKs). This induction activates decisive transcription factors and leads to the expression of matrix metalloproteinases (MMPs) such as MMP1, MMP3 and MMP9 4, 5. Among these MMPs, active MMP1 in dermal fibroblasts induces collagen fragmentation and functional alterations that resemble aged human skin. It is still unclear what events occur upstream of ROS generation during the progression of skin ageing.

NADPH oxidases are proteins that transfer electrons across biological membranes. In general, NADPH oxidases are thought to generate superoxide at the plasma membrane, nucleus and endoplasmic reticulum. The superoxide at the plasma membrane is released into the extracellular space, where it is converted into hydrogen peroxide. The biological function of NOX enzymes is to generate ROS 6, 7, 8. ROS produced by NOX proteins Nox1‐5 and Duox1/2 are now recognized to have essential roles in the physiology of the brain, immune system, vasculature, influenza virus infection and digestive tract, as well as in hormone synthesis 6, 7, 9, 10, 11. Especially, NADPH oxidase‐2 and 4 are key regulators of human dermal fibroblasts (HDFs) 12, 13.

The tumour suppressor PTEN dephosphorylates the lipid second messenger phosphoinositol 3,4,5‐trisphosphate (PIP3), an enzymatic product of PI3K, and negatively regulates survival signalling mediated by PI3K/protein kinase B/Akt (PI3K/PKB/Akt) 14, 15. NADPH oxidase is closely linked to PI3K signalling 16, 17. PKC, a downstream molecule of PI3K, is essential for NADPH oxidase‐mediated superoxide generation 16, 18. However, the role of PTEN and NOX4 in replicative ageing‐mediated MMP expression has not been determined. Therefore, we investigated the effect of PTEN on replicative‐induced MMP1 expression by increasing NOX4 and ROS generation. In this study, we demonstrate that PTEN suppresses replicative‐aged MMP‐1 expressions in replicative‐aged HDF cells. These effects are a direct result of decreased ROS production *via* the inhibition of NOX4 expression.

## Materials and methods

### Culture of HDFs

HDFs were purchased from GIBCO (Invitrogen, Carlsbad, CA, USA ). The dermal fibroblasts were cultured in high glucose DMEM with 10% foetal bovine serum and 1% antibiotics at 37°C in a 5% CO_2_ incubator. The cells were sub‐cultured in an atmosphere of 5% CO_2_ at 37°C by passaging them at a ratio of 1:4 in regular intervals. At later passages, the splitting ratio was reduced to 1:3 and 1:2. Cells were passaged such that the monolayers never exceeded 70–80% confluence. Population doublings (PD) were estimated using the following equation: n = (log10F−log10I)/0.301 (with n = population doublings, F = number of cells at the end of one passage, I = number of cells that were seeded at the beginning of one passage). The senescent status was verified by in situ staining for SA‐β‐galactosidase. At PD 55, 90–100% per cent of the cells stained positive for SA‐β‐galactosidase.

### Western blot analysis

After washing cells with PBS and harvesting, cell pellets were lysed with ice‐cold Mammalian Protein Extraction Reagent (M‐PER, Pierce Biotechnology, Rockford, IL, USA). The samples were separated using SDS‐PAGE electrophoresis.

### Quantitative real‐time PCR

Total RNA was extracted from cells using a FastPure™ RNA Kit (TaKaRa, Shiga, Japan). The cDNA was synthesized from 1 μg total RNA using a PrimeScript™ RT reagent Kit (TaKaRa). The mRNA expression levels of experimental genes and glyceraldehyde 3‐phosphate dehydrogenase (GAPDH) were determined using the ABI PRISM 7900 sequence detection system and SYBR Green (Applied Biosystems, Foster City, CA, USA). To control for variations in mRNA concentration, all results were normalized to the expression level of housekeeping gene GAPDH. Relative quantitation was performed using the comparative ∆∆Ct method according to the manufacturer's instructions.

### Quantification of intracellular ROS

The intracellular concentration of ROS in HDFs was measured using an oxidation‐sensitive fluorescent probe dye, DCF‐DA. DCF fluorescence was detected using a FACStar flow cytometer (Becton Dickinson Bioscoences, Fullerton, CA, USA).

### Vector for ectopic expression of PTEN

To generate adenoviral constructs, the PTEN entry vector (pENTR‐*PTEN*, Invitrogen) and a control entry vector (pENTR‐*GUS*) were recombined with pAD/CMV/V5 (Invitrogen) using LR Clonase II (Invitrogen) to generate pAD/CMV/*GUS*/V5 or pAD/CMV/*PTEN*/V5, respectively. Confluent HDF cultures were infected with recombinant adenovirus at a concentration of 1.0 plaque‐forming units (pfu) per cell for 72 hrs.

### RNA interference

NOX4‐specific small interfering RNA (siRNA) and negative control siRNA were purchased from BIONEER (Daejeon, Korea). Transfection of HDFs was performed according to the manufacturer's protocol (Amaxa GmbH, Cologne, Germany). Briefly, cells (3 × 10^5^) were harvested and re‐suspended in 100 μl of HDF Nucleofector Solution. The cell suspensions were mixed with 100 pmol siRNA, placed in a sterile electroporation cuvette and subjected to program P‐020 with the Nucleofector II (Amaxa GmbH).

### Statistical analysis

Statistical analysis was performed using analysis of variance and Duncan's test. *P*‐values <0.05 were considered statistically significant.

## Results and Discussion

**Figure 1:**
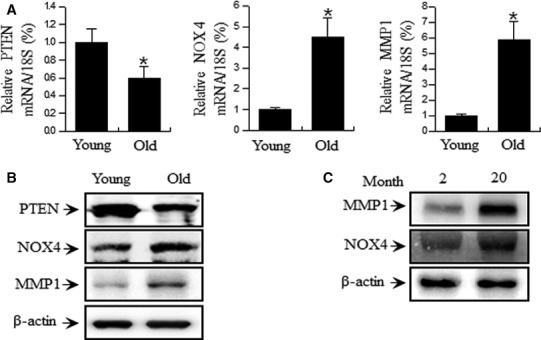
Replicative senescence‐dependent increase in PTEN, NOX4 and MMP1 levels in HDF cells. (**A**) HDFs were analysed by real‐time PCR for PTEN, NOX4 and MMP1 mRNA levels in Young (17 PD) and Old (55 PD) cells. (**B**) HDFs were analysed by Western blotting for PTEN, NOX4 and MMP1 protein levels in Young and Old PDs of HDFs. Each value represents the mean ± S.E.M. of three independent experiments. *P < 0.01 vs. Young. (**C**) Aged mouse skin tissue showed increased NOX4 and MMP1 levels. Protein expression levels of NOX4 and MMP1 in skin tissue of Young (2 months) and Old (20 months) mice were analysed by Western blotting.

Skin cells in the human body have limited replication potential. This is reflected *in vitro* by the finite replicative lifespan of cultured cells termed ‘replicative cellular senescence’*,* which has been proposed as an experimental model for human ageing 19. We also generated an *in vitro* skin ageing model by replicative sub‐culturing of HDF cells isolated from foreskin. ROS, by‐products of normal cellular oxidative processes, increase as cellular senescence progresses 20, 21 and have been demonstrated to contribute to skin cell ageing and senescence 22. In addition, ROS play a major role in skin ageing by inducing chronological ageing and photoaging 23. MMPs are responsible for the degradation or synthesis inhibition of collagenous extracellular matrix in connective tissues 24. Collagen represents the main component of dermal connective tissue extracellular matrix, and its concentration decreases in chronological ageing and photoaging. However, it is still unclear which signalling pathway upstream of ROS induces MMP expression during the progression of skin ageing. Accordingly, in this study, we investigated the ROS generation signalling pathway in replicative‐aged skin cells. Previous reports have demonstrated that increasing ROS levels in HDFs lead to the upregulation of MMP expression 25, 26. Therefore, in this study, we confirmed that the expression of MMPs increased in replicative‐aged HDF cells (Fig. 1A and 1B) and an aged mouse model (Fig. 1C).

The biological function of NOX enzymes is to generate ROS 6, 7, 27. In addition, NOX is closely linked with PI3K signalling 16, 17. PKC, a downstream molecule of PI3K, is essential for superoxide generation by NOX 16, 18. We therefore investigated to PTEN and NOX4 expression in replicative‐aged HDF cells. PTEN decreased in replicative‐aged HDF cells (Fig. 1A and B), consistent with our published results 28. And NOX4 increased in replicative‐aged HDF cells (Fig. 1).

**Figure 2:**
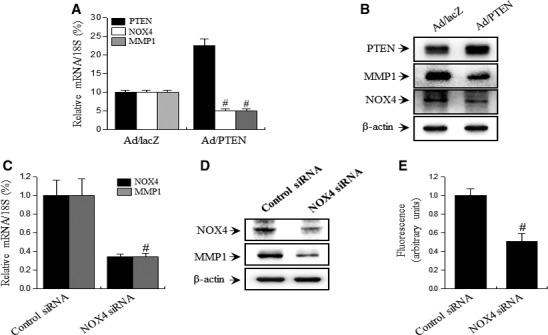
Effect of PTEN and NOX4 on replicative‐induced MMP1 expression in HDFs. Replicative‐aged HDFs (55 PD) were cultured in standard medium and infected with Ad/LacZ or Ad/PTEN. The levels of NOX4 and MMP1 expression after PTEN gene transfer or transfected with NOX4‐targeting siRNAs were analysed by real‐time PCR (**A** or **C**) and Western blotting (**B** or **D**). (**E**) In transfected replicative‐aged HDF cells with NOX4‐targeting siRNAs, ROS was measured by flow cytometry using DCFH‐DA. Each value represents the mean ± S.E.M. of three independent experiments. ^#^ *P* < 0.01 vs. Ad/lacZ or control siRNA.

Next, to investigate that the association of PTEN and NOX4 expression as upstream transduction molecules on MMP expression, we transfected senescent cells with an adenoviral vector containing PTEN gene (Ad/PTEN) or NOX4 siRNA. PTEN overexpression in replicative‐aged HDF cells abolished NOX4 and MMP1 expression (Fig. 2A and B). Additionally, NOX4 downregulation in replicative‐aged HDF cells abolished ROS level and MMP1 expression (Fig. 2C and E). These results suggest that increases of MMP1 expression by replicative‐induced ROS are related to changes in PTEN and NOX4 expression.

In conclusion, we demonstrate that .inhibition of MMP1 expression through PTEN overexpression is caused by the reduction of NOX4‐mediated ROS production. This underlines the important role of PTEN/NOX4 in replicative‐induced MMP1 expression and suggests that the regulation of NOX4/ROS by PTEN may be valid therapeutic target in replicative skin ageing.

## Conflict of interest

The authors confirm that there are no conflict of interests.
